# An Analysis of the Affordability of Harvard, Mediterranean and DASH Eating Patterns for Individuals Enrolled in the Supplementary Nutrition Assistance Program (SNAP)

**DOI:** 10.3390/nu17213480

**Published:** 2025-11-05

**Authors:** Daniel C. Knudsen, Angela M. Babb, McKenna R. Conway, Danni L. Beck

**Affiliations:** 1 Ostrom Workshop, Indiana University, Bloomington, IN 47408, USA; 2 Independent Scholar, Bloomington, IN 47403, USA; angebabb47@gmail.com; 3 Department of Geography, Indiana University, Bloomington, IN 47405, USA; mcroconw@iu.edu (M.R.C.); dannbeck@iu.edu (D.L.B.)

**Keywords:** Supplementary Nutrition Assistance Program, Thrifty Food Plan, Harvard Healthy Eating Pattern, Mediterranean Eating Pattern, Dietary Approaches to Stop Hypertension

## Abstract

Background/Objectives: In FY 2024, 12.3% of the U.S. population was enrolled in the Supplementary Nutrition Assistance Program or SNAP, the principal food and nutrition program of the U.S. government. Herein, we analyze the cost of the Harvard (HHEP), Mediterranean (MED) and Dietary Approaches to Stop Hypertension (DASH) eating patterns (EPs) to ascertain if they are affordable with the maximum allowable SNAP benefit. Methods: We utilize the 2021 Thrifty Food Plan (TFP) framework to analyze the cost of each of these alternative EPs across 15 age/sex groups. Results: We find that the MED and DASH EPs’ costs typically exceed the daily maximum SNAP benefit for those consuming more than 2100 calories. Conclusions: Our result suggests that reliance on fluid milk, allowance of more refined grains, starchy vegetables, red meat, added sugar and sodium and indifference toward food quality and freshness when calculating the maximum SNAP benefit reduces its cost in comparison to diets less reliant on fluid milk and more reliant on whole grains, fish, nuts, and fresh fruits and vegetables, a commonality shared by the HHEP, MED and DASH EPs. Health-centered alternative diets such as MED and DASH cannot be purchased with maximum allowable allocations of SNAP benefits, meaning that a poverty tax is placed on SNAP beneficiaries.

## 1. Introduction

During fiscal year (FY) 2024, approximately 41.7 million Americans, representing 12.3% of the U.S. population, participated in the Supplemental Nutrition Assistance Program (SNAP), which is the primary food and nutrition initiative of the federal government [1]. The level of SNAP benefits in any given year is a function of the national cost of a pre-determined market-basket of food in June of each year and an every-five-year analysis of the cost of a healthy and nutritious diet entitled the Thrifty Food Plan (TFP) [2]. Several controversies surround the TFP. These include aspects of the modeled eating pattern [3], the constraints and their relaxation during modeling [4,5], the adequacy of SNAP benefits [6,7,8], time and resources to create meals [9,10], the amount of food waste included in the model [11] and the model form itself [12,13].

In this article, we explicitly examine the first of these issues—aspects of the modeled eating pattern. The eating pattern modeled in the 2021 TFP (hereafter referred to as MyPlate) is only one of several deemed by the National Institutes of Health to meet the 2020–2025 Dietary Guidelines for Americans (DGA). In addition to MyPlate, DGA-compliant eating patterns also include a modification of the Mediterranean (hereafter MED) pattern [14]. Previous versions of the DGA have also included the Dietary Approaches to Stop Hypertension (hereafter DASH) eating pattern [15]. In this analysis we have also included the Harvard Healthy Eating Plan (HHEP), as it is widely researched, has been influential in nutritional circles and provides a framework that lies between MyPlate and the MED eating patterns [16].

We examine four questions concerning these eating patterns (hereafter EPs). First, is each of these EPs feasible for the fifteen age/sex categories modeled by the TFP? Second, if not, what modifications are necessary to ensure feasibility? Third, to what extent are these diets affordable for people receiving SNAP benefits? Given their status as DGA-compliant EPs, SNAP participants should be able to access these providers, as doing so can improve cardiovascular health, reduce chronic disease incidence, and lower healthcare costs. Fourth, how close do each of these EPs match current consumption for the age/sex group? This last question speaks to the degree to which those receiving SNAP must alter their diet from U.S. norms to attain a particular EP. We begin with an examination of the TFP before turning to an examination of the individual EPs and the rationale for their use.

## 2. The Origin of the TFP and Its Continuing Problems

The USDA has produced food plans since 1894, but the TFP originated in 1975 as the consequence of a lawsuit that claimed that the level of monetary benefit provided by Food Stamps (later the Supplemental Nutrition Assistance Program or SNAP) could not cover a healthy and nutritious diet [4].

In all applications of the TFP by USDA, cost has been a primary concern, if not *the* primary concern, in that the TFP has always had the minimal cost of a palatable, healthy and nutritious diet as its central theme (see also [17]). Despite this, most analyses of the TFP suggest that it fails this crucial test. Babb [4,18] notes that the TFP is based on unrealistic ideas –assuming people have several hours every day to cook from scratch and access to inexpensive healthy food selections—and, despite recent reforms to the system, USDA continues to prioritize cost-cutting over equity, nutrition and dignity for food-insecure individuals. De la Rosa and Chen [7] note that households that are SNAP beneficiaries tend to run out of SNAP benefits twenty-one days into the month, forcing them to rely on food pantries for the remaining days of the month.

The 2021 Thrifty Food Plan (TFP) reevaluation raised the cost for a reference family by 21.03%, marking the first increase not limited by cost neutrality. It reflects updated food prices, dietary guidelines, and consumption patterns, aiming to better support a healthy diet on a budget. The plan now informs higher SNAP benefits and is supposed to be reevaluated every five years by law. This increases the flexibility of households to make appropriate and practical food choices within the plan’s nutritional and cost framework [2].

Leung and Wolfson [19] found that, despite the recent increase in SNAP benefits following the 2021 TFP re-evaluation, there were no dramatic increases in food security, diet quality or mental health among SNAP beneficiaries. Food insecurity continued to be high and unchanged for SNAP participants, while nonparticipants showed little improvement. SNAP beneficiaries continued to have poor diet quality and high levels of stress, anxiety and depression versus non-participants. Some SNAP beneficiaries did show small increases in their ability to buy healthy food choices, but rising food prices and inflation largely offset any increase in benefits. Similarly, Babb et al. [8] found that, while COVID-19-pandemic-related increases improved benefit adequacy for some households, one quarter of households still found the benefits insufficient, with benefits lasting only 13 days a month.

In addition to cost considerations, issues with the assumption of universal applicability of MyPlate remain. Cohen et al. [20] imply that MyPlate may not be affordable and may be unhealthy and environmentally unfriendly. They recommend an EAT–Lancet guideline-based diet, similar to the MED EP outlined below, that includes more nuts, vegetables, beans, nuts, whole grains and seeds than MyPlate. They note that the resulting cost of this alternative diet is almost the same, but that people who require more calories may not be able to afford it. The authors suggest that the USDA consider additional support for individuals with greater caloric needs. Others have criticized MyPlate for failing to address lactose intolerance [4], relying too heavily on refined grains and starches [21], and reflecting consumed rather than recommended sodium levels.

## 3. The Harvard, Mediterranean and DASH EPs

While there is widespread consensus on the energy, macronutrient and micronutrient requirements for a healthy and nutritious diet, there is less consensus on the actual eating pattern (EP). This lack of consensus has led to a wide variety of alternatives to MyPlate, some of which are specifically acknowledged by name within the DGA [14]. Each of these EPs is provided as an alternative to MyPlate, and each is typically less reliant on fluid milk, is more focused on whole grains, explicitly acknowledges the importance of food quality and freshness, and limits red meat, added sugar and sodium.

*The Harvard Healthy Eating Pattern (HHEP)* is a response to MyPlate that promotes balanced eating by recommending half a plate of fruits and vegetables, a quarter of whole grains, and a quarter of healthy proteins like fish, beans and nuts, using healthy oils, drinking primarily water, tea or coffee, limiting dairy and avoiding sugary foods and drinks [16,22,23,24,25,26]. Following this EP can help reduce the risk of chronic disease by promoting weight-loss, long-term health and longevity [22,23]. Studies suggest the HHEP may boost longevity by up to 20% and reduce the risk of chronic diseases [22,23,25]. A 36-year study found that following any of the four healthy eating patterns presented here—especially the Harvard Healthy Eating Pattern (HHEP)—was linked to a 20% lower risk of premature death and reduced rates of cancer, respiratory diseases, type 2 diabetes and cardiovascular diseases when compared to average consumption [24,26]. The HHEP offers a flexible framework tailored to support diverse health needs and can be implemented gradually through small, sustainable changes, making it an accessible, sustainable approach to healthy eating for people of all income levels [23,24].

*The Mediterranean (MED) Eating Pattern*, also known as the Blue Zone diet, is an EP that is rooted in traditional Southern European eating patterns. The MED EP is rich in healthy fats, plant-based foods, olive oil and a moderate intake of fish, poultry, dairy and red wine while limiting red meat and processed foods [27,28]. Eating fresh food and sharing meals with others is an important part of the lifestyle and thus the MED EP [27,28]. The EP also supports environmental sustainability and is widely adaptable [27,29]. Evidence suggests that the MED EP reduces the risk of chronic diseases and improves population health [27,29]. Promoting the MED EP (also known as the MedDiet) could be a key strategy in preventing chronic diseases and improving population health [29]. Specifically, the MED EP reduces the risk of heart disease, cancer, and cognitive decline [27,30]. The EP’s health benefits come from the inclusion of bioactive compounds and antioxidants and their anti-inflammatory properties [27]. Research on the MED EP has also considered why, given the benefits to health of the EP, relatively few people adhere to it, even among people in Mediterranean countries [31]. Findings suggest that adherence to the MED EP is highest among individuals living in wealthier, urban and coastal areas. Those who are older, more educated, have higher incomes, do not smoke and exercise regularly are also more likely to follow this pattern [32]. Adherence is shaped by financial, cultural, cognitive and lifestyle factors, including health awareness, social support and the availability of adaptable food options [31]. Common barriers include cost, perceptions about fat content, limited access, lack of knowledge and cultural or climate mismatches [31]. Adoption of the EP differs across racial and ethnic groups in the U.S. because of differing food access, cultural food practices, affordability and palatability [30]. Simple, isocaloric food substitutions, especially those that include honey–food pairings, have been shown to increase Mediterranean-Style Dietary Pattern (MSDP) adherence [33].

*The DASH EP (Dietary Approaches to Stop Hypertension)* is the EP promoted by the National Blood, Heart, Lung and Blood Institute of the National Institutes of Health, and, in a slightly different form, the American Heart Association [15]. The DASH EP is rich in fruits, vegetables, whole grains, lean meats and low-fat dairy products and is highly effective at lowering blood pressure and improving overall health. Despite strong scientific evidence, the emphasis on increased fruit and vegetable consumption can feel overwhelming for people. Experts suggest making small changes, adding one healthy change at a time, and note that the DASH EP is flexible and can be easily adjusted to individual needs [34]. In studies, DASH-like EPs that limit sodium and added sugars when combined with a restricted caloric intake have been found to improve general cardiometabolic health regardless of meat intake [35]. These findings have led to initiatives such as the National Salt and Sugar Reduction Initiative (NSSRI), a public–private partnership, which argues that lowering salts and sugars in foods could prevent millions of cases of heart disease and diabetes, save billions in healthcare costs, improve quality of life and become cost-saving within nine years. It would also help reduce health gaps between different racial, income, and educational backgrounds and still be effective even if only half of the food industry follows NSSRI guidelines [36].

*Cross-EP Analyses* show that compliance with the MED and DASH EPs is related to reduced cardiovascular mortality, especially among people with hypertension and coronary heart disease. Those affected by food insecurity alone show higher all-cause mortality, while SNAP enrollment is associated with greater mortality but not cardiovascular mortality. Food security is a noticeable predictor of compliance with the MED EP, and SNAP enrollment greatly improves compliance with the DASH EP within the food-insecure population. Non-adherence to the DASH EP is related to increased mortality in people with hypertension and coronary heart disease, while non-adherence to the MED EP is linked with higher all-cause mortality in the population regardless of food security status. Poor diet quality may explain why food insecurity leads to higher death rates [37].

An important aspect of the Harvard, MED and DASH EPs is sodium reduction. Sodium is essential but generally overconsumed in the U.S., leading to higher blood pressure and heart disease risk. Research supports limiting intake to 2300 mg per day or less. Reducing sodium intake lowers blood pressure, even in healthy people, and increasing potassium helps counterbalance sodium’s effects. Most sodium comes from processed foods and eating outside the home; thus, cooking fresh foods at home and using herbs and spices can help. Yet, time to cook is a major barrier for households experiencing low incomes and food insecurity [38]. A balanced diet, like that provided by the MED EP, supports heart health with less sodium and more potassium [21]. Regarding weight loss as a target of diets, sustainable weight loss comes from long-term healthy habits, not short-term fad diets. A balanced diet, such as that provided by the HHEP, MED or DASH EPs, supports weight loss and overall health. Intermittent fasting and following a ketogenic diet may help some people, but they are not equally sustainable in the long-term [39]. We did not model any strictly vegetarian EPs because previous research has shown that they are obtainable within the maximum SNAP benefit [13].

## 4. Materials and Methods

Generally, diet models aim to minimize the distance between the allocated diet and current consumption as constrained by lower and upper bounds of necessary micronutrients, macronutrients, minerals, fatty acids, energy and eating patterns (so-called “plates” or “pyramids”) stipulating the proportions of fruits, vegetables, grains, dairy products, added sugars and proteins. The principal differences between the Thrifty Food Plan (TFP), the Harvard Healthy Eating Pattern (HHEP), the Mediterranean Eating Pattern (MED) and the DASH Eating Pattern (DASH) are the differing eating patterns and dissimilar treatment of fatty acids, added sugars and sodium. These differences are reflected in the diet model constraints, both lower and upper bounds (Table 1). In this research, we compare the feasibility, costs and deviation from current consumption of the TFP, Harvard, MED and DASH eating patterns for 15 age/sex groups. We also note other differences where warranted.

The TFP assumes that each age/sex group is moderately active. In the interest of comparability across EPs, we retain that assumption in this article as well. The resulting values from the energy constraint for each age/sex category are provided in Table 2 [2].

### 4.1. The Model

We utilize the 2021 TFP model [2] to analyze the various eating patterns. The model includes five major components: an average consumption vector specific to each age/sex group, a matrix indicating the contribution of each foodstuff to energy, micronutrient, macronutrient, mineral and eating pattern requirements, a vector of lower bounds to the energy, eating pattern, mineral, micronutrient and macronutrient requirements for the age/sex group, a vector of upper bounds to the energy, eating pattern, mineral, micronutrient and macronutrient requirements for the age/sex group, and a solution vector. The solution vector is a diet that most closely matches the consumption vector and satisfies the energy, micronutrient, macronutrient, mineral and eating pattern constraints. Following the procedure used in the 2021 TFP, the cost of a given diet is determined only after an allocated diet is determined to be feasible. Formally, our model solves the following mathematical programing problem:

MIN Z = ∑_i_ B_i_(X_i_ − T_i_)^2^, (1)

where

B_i_ = P_i_T_i_/∑_i_P_i_T_i_, (2)

and where P_i_ is the price of food group i, T_i_ is an element of the consumption vector I, and X_i_ is the corresponding amount allocated by the model of food i.

Constraints for model (1) are

L_j_ ≤ N_ij_X_i_ ≤ U_j_, (3)

L_k_ ≤ E_ik_X_i_ ≤ U_k_, (4)

and

X_i_ ≥ 0, (5)

for all food groups i, eating patterns, macronutrients, micronutrients and minerals j, and caloric restrictions k. In constraints 1–2, L and U represent lower and upper bounds, respectively.

Model infeasibility implies that not all requirements can be met by the model and that one or more lower or upper bounds will have to be relaxed to find a solution. Allocated diets with relaxed constraints typically have no drastic short-term effect on individuals, but they may have potentially serious long-term effects. The constraint sets of the various models are presented in Appendix A for clarity. Once models are determined to be feasible without a cost constraint, a cost constraint is added and incremented by USD 0.01 until feasibility is again reached. This last step determines the maximum SNAP benefit for that age/sex group. Our starting point here, given the question as to whether or not an alternative EP can be afforded given existing SNAP benefit levels, is the 2021 assigned maximum SNAP benefit level for that age/sex group [2]. Because the model uses gradient methods to find a solution, and because gradient methods do not guarantee optimal solutions, once a feasible solution is found, we utilize Excel’s built-in multi-solver to re-solve the problem 100 times to minimize the selection of a non-optimal solution.

### 4.2. Differences from the 2006 Model

The 2021 Thrifty Food Plan model differs somewhat from previous versions of the model, and we highlight those differences here, along with other important attributes of the model. This highlighting of the inner workings of the 2021 TFP is essential in the creation of comparable models for the HHEP, MED EP and DASH EP. The central difference between the 2021 TFP and previous TFPs is the use of 95 food categories in place of the previously used 45 [18]. That said, the model does find solutions for both the 95 categories and the 45 categories by utilizing a set of linkages. Thus, while the model minimizes the weighted squared distance between the model allocation and average U.S. consumption by age/sex group of the 45 food categories, it derives its cost estimates for benefit purposes from the 95-food category portion of the model. This suggests that the 2021 TFP model is a transitionary model and that 95 food categories will be used moving forward. We also note in passing that, generally, the cost of the 45 food categories as allocated by the model exceeds the cost of the allocated 95 food categories. A second major difference in the 2021 model from previous models is the use of weighted squared differences in the objective function. The rationale for the use of this function as opposed to that previously used is that squared difference functions are mathematically better behaved and thus more likely to lead efficiently to stable solutions. A third major difference in the 2021 model is that the maximum SNAP benefit is not set in advance but rather incrementally adjusted until a solution is produced. Finally, the consumption vector in the 2021 model differs from previous models in that it utilizes average U.S. consumption and not consumption of the poorest segment of the population. This reduces income bias in the TFP.

In addition to these differences, the TFP also has several other attributes, some of which are not apparent without close examination of the TFP code itself. First, the upper bounds and lower bounds for protein (meat, poultry, soy, seafood, eggs, nuts and seeds, etc.) are a function of levels of consumption. Second, for certain kinds of vegetable (dark green, red-orange, etc.), upper bounds are set at 5% above minimum consumption guidelines because few Americans meet minimum consumption requirements. Third, sodium is set as a function of consumption, not dietary guidelines. Fourth, the program incorporates a 5% food waste component. Finally, the TFP is over-constrained in the sense that binding constraints (typically those involving EPs or macronutrient intake) result in many of the micronutrient and mineral constraints being redundant. It is thus possible to solve the TFP problem with a subset of the USDA published constraints, an attribute we utilized in our own research.

### 4.3. Model Validation

We began our research by using the Freedom of Information Act to acquire the USDA TFP GAMS code and all relevant data from USDA (2021-FNS-05396-F). We then translated the TFP from GAMS into Microsoft Excel. This process provides a costless version of the TFP that can be widely distributed and allows the use of Excel’s built-in mathematical program solver. To ensure that we translated the code correctly, we performed two types of validation. We first applied the USDA’s solutions to the model to ascertain if our Excel-based model produced equivalent SNAP benefit outputs and that constraint values fell within USDA’s published constraint boundaries. We then ran the models using the TFP seeds and the TFP results as starting points. In this latter case, the models produced results that were qualitatively identical to the USDA TFP results (those foods that were allocated by the USDA TFP were allocated by our validation models) but were quantitatively different (allocated amounts were not identical). This irreproducibility stems from two causes. First, precision varies between our validation models and the TFP, with our model employing precision of the order of 1.0 × 10^−6^, while the TFP has precision of the order of 1.0 × 10^−3^. Second, the TFP as designed and implemented by USDA is a two-stage model in which the model itself interacts with the USDA Market Baskets. Our validation model has no such requirement.

### 4.4. The Alternative EPs

Our calculations for the Harvard (HHEP), Mediterranean (MED) and DASH eating patterns (EPs) utilize the TFP model but with altered EPs (Table 1). Unless otherwise noted, all alternative models utilize the same upper and lower bounds on energy, macronutrients, macronutrients and minerals as the TFP to ensure comparability.

For the HHEP, precise EPs are available for 1800 kcal and 2200 kcal [40]. EPs for the remaining age/sex categories were scaled as a function of kcal from these two precise EPs. Van Horn et al. [15] (p. 508) note that the MED EP is “inconsistently defined” [and] “widely applied.” We utilize MED EPs from Cleveland Clinic [41], Mayo Clinic [42], the US Veterans Administration [43], the Queensland provincial government [44] and the 2020–2025 DGAs [14] to define the upper and lower bounds for the MED EP. Lower bounds were set as the lowest value found from these five sources; upper bounds correspond to the highest value from these five sources. The resulting bounds tend to be wide, with the energy constraint typically providing the binding constraint for the various age/sex categories for the EP. For the DASH EP, eating pattern boundaries were available for the specific age/sex categories [15].

For each of the alternative diets, sodium (Na) was returned to the recommended amounts and not based on actual consumption levels (Table 1). While the Harvard EP sets starchy vegetable consumption to zero [40], the MED EP incorporates starchy vegetable consumption into the total grain consumption bounds [45]. The DASH EP was only applied for ages/sex categories 14+ as hypertension is rarely diagnosed in individuals below 14 years of age [46].

## 5. Results

We begin by considering whether or not each of the models is feasible without the relaxation of at least some of the model constraints (Table 3). We note that for all models in the age/sex category CH_1 (children from birth until their second birthday), we follow the USDA practice of relaxing the protein and added sugar constraints. These models are feasible for all modeled diets, given this relaxation. We also follow USDA practice in that constraints on selenium (SEL) and vitamin D (VIT_D) are relaxed in all models, and the lower bound on TOCPHA (tocopherol) is set to 85% of the lower limit of the daily requirement.

Examining the different diets, it is apparent that, given the above caveats, the TFP (MyPlate) is feasible for all age/sex groups. The HHEP is also feasible for all age/sex groups, except for M_20–50, for which the prohibition against starchy vegetables must be relaxed by 0.002 cups (0.47 mL).

The MED EP, like the HHEP diet, is feasible for all but one age/sex group. For the CH_9–11 age/sex group, the dairy constraint must be relaxed by 0.74 cups (175 mL) [47].

The DASH EP, unlike the HHEP and MED EP, is infeasible for three age/sex groups. In the case of F_20–50, the sodium upper bound must be relaxed by 267 mg, while for M_14–19, the lower bound on oil must be reduced by 24 g. For M_20–50, the lower bound on oil must be reduced by 1 g and the upper bound on nuts, seeds and soy must be relaxed upward by 2.52 ounces (71.44 g).

We next turn to a consideration of costs. Costs were calculated on feasible models so that the relaxations necessary for the solution noted in Table 3 were in place when costs were determined. Generally, all HHEP, MED and DASH EPs can be obtained at the cost of the TFP (MyPlate) (Table 4). The notable exception to this rule involves the higher energy (as measured in kcal) versions of the MED and DASH EPs, a result first noted by Cohen et al. [20]. The MED EP cost exceeded the TFP diet cost in 4 of the 15 age/sex groups. The extra expense of the MED EP averaged USD 1.63 per day. This finding corresponds to that of Tsofliou et al. [31] and Chen et al. [32], who found correspondence between MED diet adherence and wealth. The extra expense of the DASH EP averaged USD 2.37 per day.

A final consideration is the deviation between current consumption and the diets allocated by the model as measured by the value of the objective function. Table 5 shows that the TFP (MyPlate EP) best captures current (2021) consumption for age/sex categories Child_6–8, Female_12–13, 14–19, 51–70 and Male_12–13, 14–19, 20–50 and 51–70. The MED EP best captures current consumption for Child_1, 2–3, 4–5 and 9–11, Female_20–50 and 71+. The HHEP best captures current consumption for Male_71+, while the DASH EP consistently deviates furthest from current consumption.

Given constraint relaxation to obtain solutions, we must be cautious in inferring much from this. Still, it is interesting that actual consumption in the age/sex categories Child_6–8, Female_12–13 and Female_14–19 is best modeled by the MyPlate EP and that the MyPlate EP is a close second for Child_9–11. This may be related to the adherence of U.S. school lunch programs to the DGA. Similarly, the fact that current consumption by Female_20–50 and Female_71+ is closely aligned with the MED EP may be related to the lower incidence of heart disease in females in the U.S. [48].

## 6. Discussion

Prior to drawing conclusions, it is important to consider the limitations of our approach, all stemming from the limitations of the official TFP model itself, as devised by the USDA. First, use of the TFP as a framework for modeling requires precise upper and lower bounds on EPs, energy, macronutrients, micronutrients and minerals. While dietary guidelines for energy, macronutrients, micronutrients and minerals are generally agreed-upon and thus largely consistent across the TFP, HHEP, MED and DASH EPs, foods are not. For example, TFP (MyPlate) treats potatoes as a starchy vegetable, while the MED EP treats potatoes as a grain. The disagreement concerning foods is exacerbated by the separation within the TFP of dietary guidelines into 15 age/sex groups. In a sense, our methodology forces a rigidity onto the HHEP, MED and DASH EPs that does not exist in practice. Despite this, we would justify our method of partitioning these EPs by age/sex groups as necessary for our analysis, an analysis that, in turn, has genuine policy implications for food insecurity and public health within the U.S.

A second limitation stems from the way that the TFP framework clusters foods into 95 food categories. This collapse of food categories corresponds to a collapse of nutrient profiles and food prices. This is significant any time an EP substitutes one item in a category for another. For example, fluid cow’s milk and yogurt are in the same food category. In the MED EP, fluid milk is restricted in favor of yogurt. However, the price of milk is substantially lower than yogurt, and this is not fully reflected in the price of that food category. This leads to an underestimation of the cost of substituting yogurt for milk. Alternatively, the four food categories for meat include beef, pork and lamb despite their considerable price difference [49].

Third, the official TFP relaxes constraints on selenium and vitamin D and utilizes a modified constraint on TOCPHA, a strategy we have followed here to ensure comparability. In addition to these relaxations, we have had to relax additional constraints on foods within certain eating patterns to find solutions. This is especially the case for the DASH EP but is true to a lesser extent for the HHEP and MED EPs. This complicates our ability to model nutritionally adequate diets and to test the actual affordability of said diets [5].

Fourth, we assume the current consumption pattern is accessible to all, yet we know that not everyone has access to the foods they prefer [18]. Many people do not have the time or other resources to buy, store, prepare and cook most of their food from scratch, contrary to an ongoing assumption underlying the TFP and an explicit component of alternative health-centered diets (as represented by the HHEP, MED and Dash EPs) that promote whole foods over processed options. Many people live in areas demarcated by food apartheid [50], where nutritious, whole foods are not available or are otherwise inaccessible.

A final limitation concerns the use of the TFP framework itself. In using this framework, we do not wish to suggest that reforming the TFP will enable an equitable calculation of food needs or an allocation of sufficient food assistance. Food is more complex than nutrients and individual physiological needs. Our actual food needs include social, cultural and ecological needs as well, which cannot be adequately reflected within the TFP framework. For example, the MED EP is predicated on eating as a group and taking time as part of mealtime to converse with others; these conditions are obviously not included in our analysis. We also recognize the TFP is a tool first created to rationalize an insufficient Food Stamp Program and that it perpetuates a long outdated and inaccurate poverty calculation [51].

In addition to addressing its limitations, this research could be expanded in at least three ways. First, all models used here adopt the standard TFP practice of not constraining Vitamin D and using a lower bound of 85% of the minimum daily requirement of TOCPHA. It would be interesting to ascertain the effect on cost of meeting the minimum daily requirements of these two micronutrients. Second, while vegetarianism has been analyzed at least implicitly within a TFP framework elsewhere [13], veganism has not been considered within the TFP framework. Third, given our findings, the relationship between the SNAP and Food as Medicine programs, especially those that might target at-risk, high-caloric-need individuals (i.e., those employed in active service jobs, construction or agriculture) merits examination.

## 7. Conclusions

Ambrosini et al. [37] note that individuals affected by food insecurity have higher all-cause mortality. While those participating in SNAP and following the DASH EP have reduced rates of cardiovascular mortality, non-adherence to the MED EP is linked to higher all-cause mortality in the population regardless of food security status [37].

We find that SNAP benefits are insufficient to afford higher caloric MED EPs and nearly all DASH EPs. The MED and DASH diets have both been proven to increase lifespan and lessen the incidence of chronic disease. The fact that these alternative diets are not affordable with SNAP suggests that SNAP recipients, do, in fact, effectively pay a poverty tax [52] via a higher incidence of cardiovascular mortality and all-cause mortality. Under the current federal budget, which is projected to cut USD 187 billion from SNAP (approximately 20% of current expenditure) through 2034, this tax is predicted to increase [53].

MED and DASH EPs’ costs typically exceed the daily maximum SNAP benefit for those consuming more than 2100 calories. This suggests that the TFP’s reliance on fluid milk, allowance of more refined grains, starchy vegetables, red meat, added sugar and sodium and indifference toward food quality and freshness reduces its cost in comparison to diets less reliant on fluid milk and more reliant on whole grains, fish, nuts and fresh fruits and vegetables, a commonality shared by the HHEP, MED and DASH EPs. It also suggests that food policy needs to better consider the caloric needs of individuals when setting SNAP benefit levels.

## Figures and Tables

**Table 1:** Brief comparison of TFP, Harvard, Mediterranean and Dash EPs. Recommended/allowed minimum and maximum daily intake, adult male, aged 20–50.

Food Category	TFP (MyPlate) [2]	HHEP [40] ^1^	MED [14,41,42,43,44,45]	DASH [15]
**Dairy** (cups)	3–3.88	1–2.73	0–1	2–3
**Protein** (ounces)	7–12.78	3.85–8.18	4.7–6.5	6–7
Meat (ounces)	1.3–3.86		0–0.43	
Seafood (ounces)	1.47–1.97	1.7–8.18	1–2.14	1.1–1.4
Poultry (ounces)	0–4.34		0–3	
Eggs (ounces)	0–0.86		0–1	
Nuts and Seeds (ounces)	0–2.91	1–2	0.214–0.9	
Meat, Poultry, Eggs (ounces)	0–5.43			3.7–4.7
Nuts, Seeds, Soy (ounces)	1–2			0–0.71
**Added Sugars** (kcal)	0–300	0–150	0–150	0–150
**Grains** (ounces)	10–12.56	5–10.91	3–6	6–10
Whole Grains (ounces)	2.46–12.56	5–10.91	3–6	5–10
Refined Grains (ounces)	0–5	0–5	0–3	0–5
**Vegetables** (cups)	2.88–4	2.14–4.09	1.5–5	2.5–5
Dark Green (cups)	0.36–0.46			0.21–0.36
Red/Orange (cups)	0.76–1.07			0.76–1.07
Starchy (cups)	0.96–1.14	0	0–0.5 ^2^	0.71–1.14
Other (cups)	1–1.02			0.57–1
Legumes (cups)	0.43–0.5		0.214–0.286	0.21–0.43
**Fruits** (cups)	2.5–2.7	1.5–2.73	1.5–3	2–2.5
Fruit Juice (cups)	0–1.25	0–0.5		
Whole Fruit (cups)	1.25–2			
Sodium (mg)	1500–4188	1500–2300	1500–2300	1500–2300
**Oils** (g)	44–52.2	14–28	14–56	45–75
**Fats** (g)	66.7–116.7	66.7–116.7	66.7–116.7	13–20 ^3^
18:2 Linoleic Acid (g)	16.7–33.3			
18:3 Linolenic Acid (g)	2–4			
**Energy** (kcal)	2985–3015	2985–3015	2985–3015	2985–3015

**Note:** Blanks in table denote no specific guidelines for this category and corresponding relaxed constraint. Metric conversions: 1 cup = 236.6 mL; 1 ounce = 28.36 g. ^1^ Imputed from the 51–70 age category. ^2^ Allowed but counted as grain. ^3^ Only considers solid fats.

**Table 2:** Assumed energy (kcal) by age/sex group.

Age/Sex Group	Assigned Level (kcal)	Lower Bound	Upper Bound
Child_1	1000	995	1005
Child_2–3	1400	1393	1407
Child_4–5	1600	1592	1608
Child_6–8	1800	1791	1809
Child_9–11	2200	2189	2211
Female_12–13	2000	1990	2010
Female_14–19	2200	2189	2211
Female_20–50	2200	2189	2211
Female_51–70	2000	1990	2010
Female_71+	1800	1791	1809
Male_12–13	2400	2388	2412
Male_14–19	3000	2985	3015
Male_20–50	3000	2985	3015
Male_51–70	2600	2587	2613
Male_71+	2400	2388	2412

**Table 3:** Model feasibility by age/sex group for each EP.

Age/Sex Group	TFP (MyPlate) ^1^	HHEP ^1^	MED EP ^1^	DASH EP ^1^
Child_1	Feasible ^2^	Feasible ^2^	Feasible ^2^	^8^
Child_2–3	Feasible	Feasible	Feasible	^8^
Child_4–5	Feasible	Feasible	Feasible	^8^
Child_6–8	Feasible	Feasible	Feasible	^8^
Child_9–11	Feasible	Feasible	Infeasible ^4^	^8^
Female_12–13	Feasible	Feasible	Feasible	^8^
Female_14–19	Feasible	Feasible	Feasible	Feasible
Female_20–50	Feasible	Feasible	Feasible	Infeasible ^5^
Female_51–70	Feasible	Feasible	Feasible	Feasible
Female_71+	Feasible	Feasible	Feasible	Feasible
Male_12–13	Feasible	Feasible	Feasible	^8^
Male_14–19	Feasible	Feasible	Feasible	Infeasible ^6^
Male_20–50	Feasible	Infeasible ^3^	Feasible	Infeasible ^7^
Male_51–70	Feasible	Feasible	Feasible	Feasible
Male_71+	Feasible	Feasible	Feasible	Feasible

^1^ All models have SEL, VIT_D relaxed and TOCPHA at 85% by default. ^2^ Proteins and added sugars relaxed in all models by default. ^3^ UB on starch relaxed for solution. ^4^ UB on dairy relaxed for solution. ^5^ UB on Na (sodium) relaxed for solution. ^6^ LB on oil relaxed for solution. ^7^ LB on oil and UB on nuts, seeds and soy relaxed for solution. ^8^ Analysis not conducted for this age/sex group.

**Table 4:** Daily cost of allocated diet by age/sex group at solution in 2021 in USD for each EP ^1^.

Age/Sex Group	TFP (MyPlate)	HHEP	MED EP	DASH EP
Child_1	3.15	3.15	3.15	^2^
Child_2–3	4.71	4.71	4.71	^2^
Child_4–5	5.07	5.07	5.07	^2^
Child_6–8	5.68	5.68	5.68	^2^
Child_9–11	6.55	6.55	6.55	^2^
Female_12–13	6.09	6.09	6.09	^2^
Female_14–19	6.96	6.96	6.96	7.21
Female_20–50	6.83	6.83	6.83	10.12
Female_51–70	6.34	6.34	6.34	6.34
Female_71+	7.00	7.00	7.00	7.00
Male_12–13	6.99	6.99	6.99	^2^
Male_14–19	8.78	8.78	10.02	12.31
Male_20–50	8.54	8.54	9.96	13.60
Male_51–70	7.48	7.48	9.36	9.03
Male_71+	7.16	7.16	9.15	7.71

^1^ See restrictions noted below Table 3. ^2^ Analysis not conducted for this age/sex group.

**Table 5:** Objective function values of the 45 food categories by age/sex group for each EP ^1^.

Age/Sex Group	TFP (MyPlate) ^2^	HHEP	MED EP	DASH EP
Child_1	0.521375	0.314392	0.139194	^3^
Child_2–3	0.826282	0.582789	0.112682	^3^
Child_4–5	0.675254	0.553173	0.14998	^3^
Child_6–8	0.082257	0.715283	0.1548	^3^
Child_9–11	0.285657	0.711506	0.226469	^3^
Female_12–13	0.43963	0.995321	0.577327	^3^
Female_14–19	0.613144	0.802545	0.924581	1.39915
Female_20–50	0.271691	0.709386	0.109678	1.29463
Female_51–70	0.309819	0.752144	0.507224	1.15205
Female_71+	0.175212	0.549544	0.134074	0.620579
Male_12–13	0.225503	0.801071	0.558235	^3^
Male_14–19	0.819332	0.922026	2.153009	1.162592
Male_20–50	0.532012	1.222997	3.626956	2.755024
Male_51–70	0.628271	0.870737	10.63862	1.373824
Male_71+	0.79995	0.618838	16.4763	1.405239

^1^ See restrictions noted below Table 3. ^2^ Values from our calibration of the TFP, not those of the USDA TFP. ^3^ Analysis not conducted for this age/sex group.

## Data Availability

The data utilized here can be found online in the supplemental files for the 2021 Thrifty Food Plan (https://www.fns.usda.gov/cnpp/thrifty-food-plan-2021; accessed on 11 October 2025. Relevant data can also be accessed online here: http://www.shieldsridgefarm.com/thrifty-food-plan-2021.html (accessed on 12 October 2025). The latter webpage also contains a prototypical model in Excel.

## Abbreviations

The following abbreviations are used in this manuscript:

SNAP	Supplementary Nutrition Assistance Program
TFP	Thrifty Food Plan
DGA	Dietary Guidelines for Americans
EP	Eating Pattern
HHEP	Harvard Healthy Eating Pattern
MED	Mediterranean
DASH	Dietary Approaches to Stop Hypertension

## Appendix A

**Table A1:** Brief comparison of constraints in the TFP, Harvard, Mediterranean and DASH models.

Constraint	TFP	HHEP	MED	DASH
F_JUICE	ON	ON	ON	OFF
F_TOTAL	ON	ON	ON	ON
V_DRKGR	ON	OFF	OFF	ON
V_REDOR_TOTAL	ON	OFF	OFF	ON
V_STARCHY_TOTAL	ON	ON	ON	ON
V_OTHER	ON	OFF	OFF	ON
V_TOTAL	ON	ON	ON	ON
V_LEGUMES	ON	ON	ON	ON
G_WHOLE	ON	ON	ON	ON
G_REFINED	ON	ON	ON	ON
G_TOTAL	ON	ON	ON	ON
PF_MEAT	ON	OFF	ON	OFF
PF_POULT	ON	OFF	OFF	OFF
PF_EGGS	ON	OFF	OFF	OFF
PF_SOY	ON	OFF	OFF	OFF
PF_NUTSDS	ON	ON	ON	OFF
PF_TOTAL	ON	ON	ON	ON
D_TOTAL	ON	ON	ON	ON
OILS	ON	ON	ON	ON
ADD_SUGARS	ON	ON	ON	ON
T_MEAT_POULTRY_EGG	ON	OFF	OFF	ON
T_SEAFOOD	ON	ON	ON	ON
T_NUT_SEED_SOY	ON	OFF	OFF	ON
T_WHOLEFRT	ON	OFF	OFF	OFF
CA	ON	ON	ON	ON
CHOCDF	ON	ON	ON	ON
CHOLN	ON	ON	ON	ON
CU	ON	ON	ON	ON
ENERC_KCAL	ON	ON	ON	ON
F18D2	ON	ON	ON	ON
F18D3	ON	ON	ON	ON
FASAT	ON	ON	ON	ON
FAT	ON	ON	ON	ON
FE	ON	ON	ON	ON
FIBTG	ON	ON	ON	ON
FOLDFE	ON	ON	ON	ON
FOLAC	ON	ON	ON	ON
K	ON	ON	ON	ON
MG	ON	ON	ON	ON
NA	ON	ON	ON	ON
NIA	ON	ON	ON	ON
P	ON	ON	ON	ON
PROCNT	ON	ON	ON	ON
RIBF	ON	ON	ON	ON
THIA	ON	ON	ON	ON
TOCPHA	MOD	MOD	MOD	MOD
VITA_RAE	ON	ON	ON	ON
VITB12	ON	ON	ON	ON
VITB6A	ON	ON	ON	ON
VITC	ON	ON	ON	ON
VITD	OFF	OFF	OFF	OFF
VITK	ON	ON	ON	ON
ZN	ON	ON	ON	ON
sfat_kcal	OFF	OFF	OFF	OFF
add_sugars_kcal	OFF	OFF	OFF	OFF
breakfast_kcal	OFF	OFF	OFF	OFF
